# Impact of Neutrophil-Secreted Myeloid Related Proteins 8 and 14 (MRP 8/14) on Leishmaniasis Progression

**DOI:** 10.1371/journal.pntd.0002461

**Published:** 2013-09-26

**Authors:** Irazú Contreras, Marina T. Shio, Annabelle Cesaro, Philippe A. Tessier, Martin Olivier

**Affiliations:** 1 Department of Microbiology and Immunology, McGill University, Montreal, Quebec, Canada; 2 Research Institute of the McGill University Health Centre and the McGill International Tuberculosis Centre, Montréal, Quebec, Canada; 3 Infectious Disease Research Centre, Laval University, Québec, Quebec, Canada; Hospital Universitário, Brazil

## Abstract

The myeloid-related proteins (MRPs) 8/14 are small proteins mainly produced by neutrophils, which have been reported to induce NO production in macrophages. On the other hand, *Leishmania* survives and multiplies within phagocytes by inactivating several of their microbicidal functions. Whereas MRPs are rapidly released during the innate immune response, their role in the regulation of Leishmaniasis is still unknown. *In vitro* experiments revealed that *Leishmania* infection alters MRP-induced signaling, leading to inhibition of macrophage functions (NO, TNF-α). In contrast, MRP-primed cells showed normal signaling activation and NO production in response to *Leishmania* infection. Using a murine air-pouch model, we observed that infection with *L. major* induced leukocyte recruitment and MRP secretion comparable to LPS-treated mice. Depletion of MRPs significantly reduced these inflammatory events and augmented both parasite load and footpad swelling during the first 8 weeks post-infection, as also observed in MRP KO mice. On the contrary, mouse treatment with recombinant MRPs (rMRPs) had the opposite effect. Collectively, our results suggest that rapid secretion of MRPs by neutrophils at the site of infection may protect uninfected macrophages and favor a more efficient innate inflammatory response against *Leishmania* infection. In summary, our study reveals the critical role played by MRPs in the regulation of *Leishmania* infection and how this pathogen can subvert its action.

## Author Summary

Parasites of the *Leishmania* genus have developed multiple mechanisms to subvert the immune response. Among these mechanisms are the activation of host phosphatases and inactivation of cell signaling pathways, which in turn activate the immune response. On the other hand, it has been observed that the Myeloid Related Proteins (MRPs) 8 and 14 are potent activators of some components of the immune response. In this study, we evaluated the effect of MRPs 8 and 14 on the progression of cutaneous Leishmaniasis. To do so, we used immortalized macrophages and stimulated them with MRPs before or after infection with *L. major*. We observed that stimulating macrophages with MRPs prior to infection induced NO and TNF-α production, as well as phosphorylation of MAPKs and nuclear translocation of transcription factors NF-κB and AP-1. However, when MRP stimulation was performed after infection, these effects where subverted. Moreover, using a murine model of cutaneous infection, we observed that depletion of MRPs caused increased parasite burden and bigger lesions. On the contrary, injection of recombinant MRPs directly into the lesion, considerably reduced lesion size and parasite burden. Our study suggests that MRPs could have a potential therapeutic use in the control of *Leishmania* infection.

## Introduction

Myeloid-related proteins 8 and 14 (MRPs 8/14) also known as S100A8 and S100A9 are small calcium binding cytoplasmic proteins secreted mainly by neutrophils and monocytes [1], [2]. These proteins are formed by two Ca^2+^ binding domains separated by a hinge region [3]. Although these proteins exist as homodimers, a heterodimer (MRP 8/14) is formed in the presence of calcium. Both proteins are expressed abundantly by neutrophils, being around 30 to 40% of their cytoplasmic proteins [4]. MRP 8 and 14 are not constitutively expressed by macrophages; however, expression of MRP 8 can be achieved in those cells by stimulation with LPS, IFN-γ, IL-1β and TNF-α. Interestingly, murine endothelial cells express both MRP 8 and MRP 14 following LPS stimulation [5]. Murine MRP 8 is chemotactic for neutrophils and monocytes, whereas human MRP 14 and the heterodimer MRP 8/14 are chemotactic for neutrophils, stimulate their adhesion to fibrinogen, and enhance monocyte transmigration across endothelial cells. It is also known that MRP 8 and 14 inhibit bacterial growth possibly by zinc chelation and by preventing bacterial adhesion to mucosal epithelial cells [6].

MRP 8 and 14 have been associated with a number of inflammatory diseases leading to the assumption that these molecules are involved in the body's defense against inflammation. Phagocytes expressing MRP 8 and 14 are found in a variety of inflammatory conditions, including rheumatoid arthritis, chronic bronchitis and inflammatory bowel disease [7], [8]. Moreover, Tessier and collaborators [1] reported that in the murine air-pouch model, stimulation with LPS led to an abundant recruitment of neutrophils and subsequent secretion of MRPs [2].

We have previously reported that MRP 8 and 14 play an important role in the nitric oxide (NO) modulation; a key microbicidal function of macrophages [9]. This increase was linked with augmented expression of inducible nitric oxide synthase (iNOS), at the gene and protein levels, concomitantly with ERK and JNK kinases phosphorylation and the rapid NF-κB nuclear translocation. These findings indicate that MRPs play an important role during inflammation.

Although much is known about MRPs during inflammation and inflammatory diseases; little is known about the potential role of MRPs in Leishmaniasis. Leishmaniasis (caused by parasites of the *Leishmania* genus), is a disease characterized by three main clinical manifestations; cutaneous Leishmaniasis, muco-cutaneous Leishmaniasis and the lethal if untreated visceral Leishmaniasis. *Leishmania* parasites of different species are able to abrogate the innate immune response in order to survive inside their host cell [10]. In regard of the role of MRPs in Leishmaniasis, only two reports have documented accumulation of macrophages expressing MRP 8 and 14 at the skin lesions of mice infected with *L. major* [11], [12]. They also found, that amastigotes isolated from skin lesions presented MRP 8 and 14 adhered onto their surface. However, and despite these observations, the role of these proteins during *Leishmania* infection has not been investigated.

Herein, we report the first study concerning the role of MRPs during *Leishmania* infection in a murine experimental model. More precisely, we found that MRP-primed macrophages infected by *L. major* exhibit antimicrobial activity, whereas unprimed *L. major*-infected cells were fully inactivated, showing no response to MRP stimulation. Using *in vivo* approaches, we further demonstrated that *L. major*'s capacity to recruit inflammatory cells was accompanied by MRP secretion at the site of inoculation. The use of anti-MRP antibodies in addition to blocking *Leishmania*-induced leukocyte recruitment in the air-pouch also increased mice footpad swelling and parasite load. Similarly, MRP deficient mice were found more sensitive to develop footpad swelling. Importantly, use of recombinant MRPs (rMRPs) to treat infected footpads led to significantly reduced footpad swelling and lesion development as well as a reduced parasite load. Altogether, this study provides a clear demonstration that MRPs seem to play a critical role in the control of the progression of *Leishmania* infection by modulating the innate inflammatory and microbicidal responses.

## Material and Methods

### Animals

The research involving animals in this work was carried out according with the regulations of the Canadian Council of Animal Care and approved by the McGill University Animal Care Committee (AUP#4859). BALB/c and C57Bl/6 mice were obtained from Charles River and Jackson Laboratory, respectively. MRP14 KO mice (C57Bl/6 background) were obtained from Dr. Philippe Tessier's laboratory at Laval University, QC. Canada.

### Cell culture, macrophage infection and reagents

Immortalized murine bone marrow derived macrophages B10R cell line were grown at 37°C in 5% CO_2_ in Dulbecco's Modified Eagle medium (DMEM) supplemented with 10% heat inactivated FBS (Invitrogen, Burlington ON, Canada) and 100 U/ml penicillin 100 µg/ml streptomycin and 2 mM of L-glutamine (Wisent, St. Bruno, QC, Canada). *Leishmania* promastigotes (*L. major* A2 and *L. major* luciferase) were grown and maintained at 25°C in SDM-79 culture medium supplemented with 10% FBS by bi-weekly passage. Macrophages were infected at a parasite-macrophage ratio 20∶1 with stationary phase promastigotes for the times specified in each figure legend. When cells were primed with 5, 10 and 25 µg/ml of MRPs 8/14 heterodimer were used 1 hr before infection and remained throughout the infection time. All reagents if not indicated were obtained from Sigma Aldrich (St-Louis MO, USA).

### Recombinant proteins

Cloning expression, and purification of mouse MRP 8 and 14 (S100A8/A9) were previously described [1], [2]. Briefly, mouse S100A8 cDNA was cloned into the pET28a expression vector (Novagen). Murine S100A9 cDNA was obtained by RT-PCR and cloned into the PET28a vector (Dr. Philippe Tessier's laboratory, Laval University, QC. Canada). Recombinant protein expression was induced with 1 mM isopropyl-β-D-thiogalactoside in *E. coli* HMS174 for 16 hr at 16°C. After incubation, the bacteria were centrifugated and the pellet resuspended in PBS/NaCl (0.5 M)/imidazole (1 mM) and lysed by sonication. The pellet was centrifugated and the supernatant collected. Recombinant His-Tag proteins were purified using a nickel column; S100A8/A9 bound to the column were freed from their His-Tag by incubation with 10 U of biotinylated thrombin for 20 hr at RT. Finally the proteins were passed through a polymyxin B agarose column (Pierce, Rockford, IL USA) to remove endotoxins. The lysate, contamination by endotoxins was <1 pg/µg. The proteins were kept at −80°C until further use.

### Nitric oxide measurements

B10R macrophages were plated in 12-well plates (0.5×10^6^ cells/well, in triplicates). The next day, cells were pre-treated for 24 hr with 5, 10 and 25 µg/ml of MRPs 8/14 and then infected with *L. major* (20∶1) for another 24 hr (MRP-primed-infected); or pre-infected with *L. major* for 24 hr and then stimulated with 5, 10 and 25 µg/ml of MRPs for further 24 hr (infected-MRP-stimulated). NO production was assessed by measuring the accumulation of nitrites in the cell culture medium using the colorimetric Griess reaction as previously described [13].

### TNF bioassay

B10R macrophages were plated in 12-well plates (0.5×10^6^ cells/well). Next day, cells were stimulated: MRPs alone, MRP-primed-infected or infected-MRP-stimulated. After the different times of stimulation (indicated in the figure legend) or infection, plates were centrifugated at 2500 rpm and 100 µl of supernatant were collected and added to TNF-sensitive L-929 fibroblasts [14] previously pleated in 96-well plates (3×10^5^ cells/well/100 µl), in the following day 100 µl of B10R culture supernatant were added to the L929 cells making a 2-fold serial dilution. Actinomycin D (final concentration of 2 µg/ml) was added to each well and plates were incubated 18 hr at 37°C. Next day, live cells were stained with crystal violet (0.05% in 0.1% acetic acid solution) for 10 minutes. After, plates were washed to remove excess of stain and 100 µl of 100% methanol were added to each well to elute stain from the cells. Plates were red at 595 nm. Data are expressed as unit of TNF referring to the dilution that induced 50% of L929 cell death.

### Electrophoresis Mobility Shift Assay (EMSA)

B10R macrophages (2×10^6^) stimulated with MRPs alone (1 hr), MRP-primed (1 hr)-infected (1 hr) or infected (24 hr)-MRP-stimulated (1 hr) were washed three times with PBS to remove non-internalized parasites, and processed for nuclear extraction as previously described [15], [16]. Briefly, macrophages were collected in 1 ml of cold PBS, centrifuged and pellets were resuspended in 400 µl of ice-cold buffer A (10 mM HEPES, 10 mM KCl, 0.1 mM EDTA, 0.1 mM EGTA, 1 mM DTT and 1 mM of PMSF) and incubated 15 min on ice. 25 µl of IGEPAL 10% were added, and samples vortexed for 30 sec. Nuclear proteins were pelleted by centrifugation and resuspended in 50 µl of cold buffer C (20 mM HEPES, 400 mM NaCl 1 mM EDTA, 1 mM EGTA 1 mM DTT and 1 mM PMSF).

Protein concentrations were determined by Bradford assay (Bio-Rad, Hercules CA, USA). 6 µg of nuclear proteins were incubated for 20 min at room temperature with 1 µl of binding buffer (100 nM Hepes pH 7.9, 8% v/v glycerol, 1% w/v Ficoll, 25 mM KCl, 1 mM DTT, 0.5 mM EDTA, 25 mM NaCl, and 1 µg/µl BSA) and 200 ng/µl of poly (dI-dC), 0.02% bromophenol blue and 1 µl of γ-P^32^labeled oligonucleotide containing a consensus sequence for AP-1 binding complexes (5′-CGTTTGATGACTCAGCCGGAA-3′) (Santa Cruz Biotechnology Inc, Dallas, TX, USA), NF-κB (5′-AGTTGAGGGGACTTTCCCAGGC-3′) (Santa Cruz Biotechnology Inc) and STAT1 (5′-AAGTACTTTCAGTTTCATATTACTCTA-3′). After incubation, DNA-protein complexes were resolved by electrophoresis in non-denaturing polyacrylamide gel 5% (w/v). Subsequently gels were dried and autoradiographed. Competition assays were conducted by adding a 100-fold molar excess of homologous unlabeled AP-1 oligonucleotide, or the non-specific competitor sequence for SP-1 binding (5′-ATTCGAATCGGGGCGGGGCGAGC-3′).

### Western blot

B10R cells (1×10^6^) stimulated with MRPs alone (30 min), MRP-primed (30 min)- infected (1 hr) or infected (1 hr)-MRP-stimulated (30 min) were washed 3 times with PBS and lysed with cold buffer (50 mM Tris-HCl pH 7.0, 0.1 mM, 0.1 mM EGTA, 0.1% 2-mercaptoethanol, 1% NP-40, 40 µg/ml aproptinin, 20 µg/ml of leupeptin 100 mM PMSF, and 20 mM NaVO_4_). Proteins were dosed by Bradford (Bio-Rad), and 30–60 µg of proteins were separated by SDS-PAGE, and transferred onto PVDF membranes (GE healthcare, Piskataway NJ, USA). Membranes were blocked in 5% bovine serum albumin (Wisent), washed and incubated ON with anti-phospho or total ERK, phospho or total -JNK, iNOS (Cell signaling, Ipswich, MA, USA) or β-actin. After washing, membranes were incubated 1 hr with α-rabbit or mouse HRP-conjugated antibody, and developed by autoradiography.

### Luciferase assays

To determine parasite survival inside B10R macrophages, cells were plated in 12-well plates (0.5×10^6^cells/well) and the following day they were infected with stationary phase *L. major*-LUC promastigotes (10∶1 ratio). After 6 hr of infection, the non-phagocytosed parasites were removed by washes with PBS, and samples were collected. For the second group of samples, fresh media was added and cells were incubated for another 18 hr. Adherent macrophages were collected and centrifuged 13,000 rpm×1 min. Pellets were lysed in 25 µl of 1× Cell Culture Lysis Reagent (Promega, Fitchburg, WI, USA). 20 µl of lysate were mixed with 90 µl of Luciferase Assay Reagent (Promega) and luciferase counts were determined using a Mini Lumat LB 9506 luminometer (EG&G).

### Air pouch

Air pouches were raised on the dorsum of 6 week-old BALB/c by s.c. injection of 3 ml of sterile air on days 0 and 3. On day 6, 1 ml of LPS (1 µg/ml) or 5×10^6^ parasites of *Leishmania major* in 1 ml of PBS were injected into the air pouches. At 6 hr, mice were sacrificed and air pouches were washed twice with 2 ml of PBS. Exudates were centrifuged at 1200 rpm for 5 min. Cells were counted with a hematocytometer. Characterization of leukocyte subpopulations migrated into the pouch space was performed by diff-quick staining of cytospins. In some experiments mice were injected i.p. with 4 mg of purified rabbit IgG anti-MRP8/14 16 hr before infection.

### MRP secretion in the air-pouch

To determine the concentration of MRP 8/14 (S100A8/A9) in the air pouch, ELISAs were performed as previously described in [2]. Briefly, Costar high binding 96-well plates (Corning Glass, Tewksbury MA, USA) were coated overnight at 4°C with 100 µl of purified rabbit IgG against MRP8 or MRP14, diluted in 1 µg/ml in 0.1 M of carbonate buffer, pH 9.6. The wells were blocked with PBS/0.1% Tween 20/2% BSA for 30 min at room temperature. Then the samples and the standards (100 µl) were added, and after 45-min period at room temperature, the plates were incubated with rat IgG (100 µl/well) against MRP8 and MRP14 diluted in PBS/0.1% Tween 20/2% BSA for 45 minutes. To reveal the immune complex, 100 µl/well of peroxidase-conjugated goat-anti-rat was added and incubated for 45 minutes. Next 100 µl/well of 3,3′, 5,5′-tetraamethylbenzidine substrate (Research diagnostics, Las Vegas, NV, USA) were added according to the manufacturer's instructions, and ODs were read at 500 nm. The lower limit of quantification was determined as 4 ng/ml for both MRP8 and MRP 14, and 10 ng/ml for the heterodimer. All ELISAs were tested using excess amounts of the other S100 proteins and were shown to be specific under conditions reported in this work.

### Footpad infection

*L. major* stationary phase promastigotes (5×10^6^ in 50 µl of PBS) were injected in mice's right hind footpad. Footpad thickness measurement was performed as previously described [17] for 10–12 weeks. For the group of MRP neutralization, anti-MRP 8/14 (4 µg/ml) were injected i.p. 1 day after infection and then at days 3, 6, 9, 12, 15, 18 and 21 after infection. After 8 weeks, mice were sacrificed and parasite load was measured by limiting dilution assay. For the group that received recombinant MRPs (rMPR) as treatment, mice were infected as previously described and then treated 3 times per week with 10 µg of the mix MRP8/14 in 50 µl of PBS, during the last four weeks of infection directly in the infected footpad. Thickness of the lesion was measured every week until the end of the infection and parasite load was measured as described below.

### Limiting dilution assay

Limiting dilution assay was done as previously described [18], [19] with some modifications. Briefly, after 10–12 weeks of infection mice were sacrificed. The infected footpads were disinfected and inflamed area of the pad was excised, homogenized; extracted parasites were serially diluted in a 96 well plate in duplicate. After 8 days, the number of viable parasites was determined from the highest dilution using an inverted microscopy.

### Statistical analysis

Statistically significant differences were analyzed by ANOVA followed by Tukey test using the Graphpad Prism program (version 5.0). For limiting dilution and TNF, non-parametric Mann-Whitney or Kruskal-Wallis test was used. Values of P≤0.05 were considered statistically significant. All data are presented as mean ± SEM.

## Results

### *Leishmania* infection down-regulates MRPs-inducible NO production

**Figure 1:**
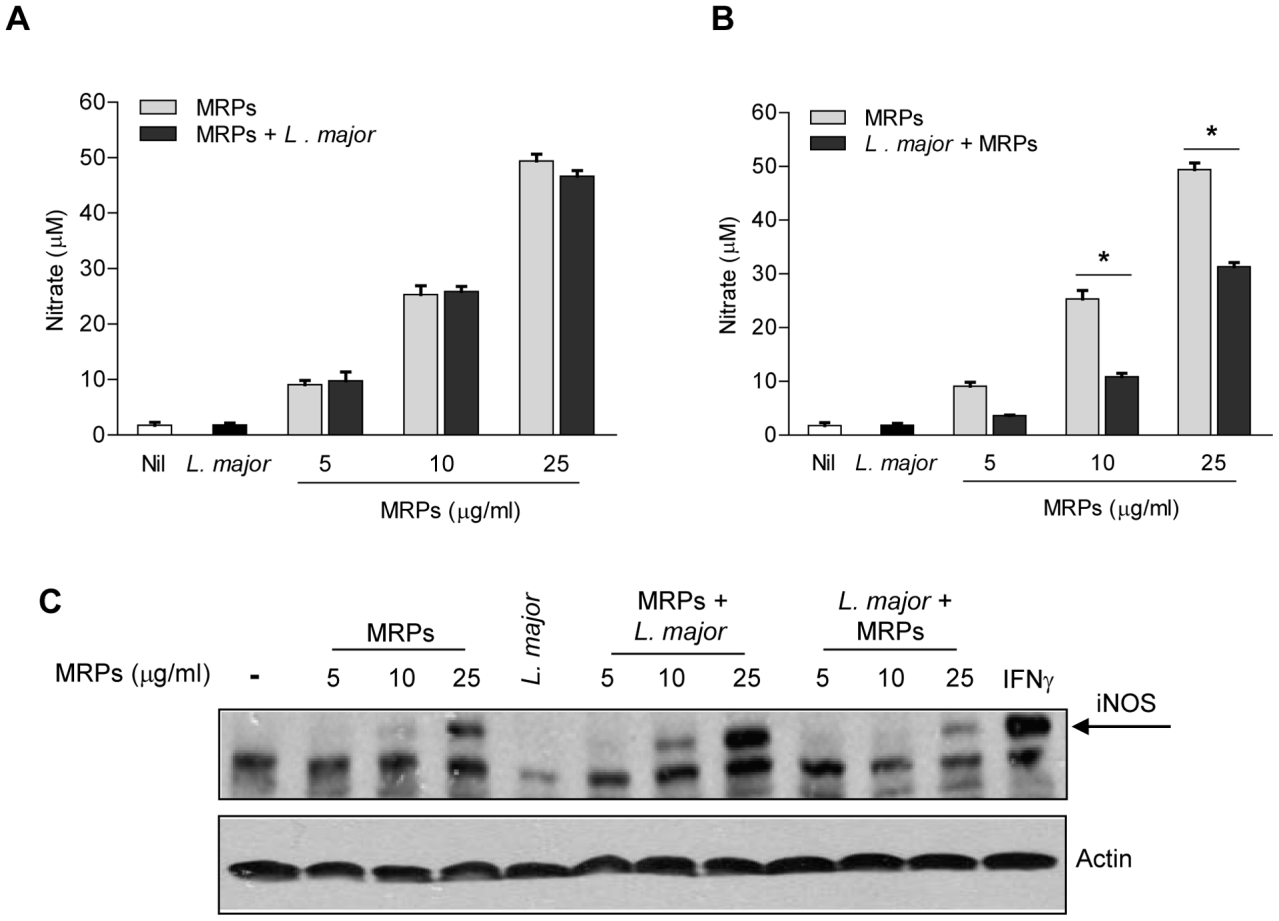
Impact of *Leishmania* infection on the production of nitric oxide (NO) and iNOS expression by MRPs 8/14. B10R macrophages were stimulated with different doses of MPRs 8/14 (5, 10 and 25 µg/ml) before (**A**) or after (**B**) of infection (24 hr) with *L. major*. NO production (after 24 hr) was assessed by measuring nitrates in the supernatants. (**C**) B10R macrophages were treated as in (**A**) and (**B**) and protein lysates were subjected to immunoblotting analysis with anti-iNOS. Equal protein loading is shown by β-actin. (*) denotes P<0.05 between the MRP-stimulated macrophages and *L. major* infected-MRP-stimulated macrophages. Mean of three different experiments is shown.

We have previously described that MRPs induce NO in murine macrophages [9]. Confirming and extending these data, we observed that increasing concentrations of MRPs 8/14 (5, 10 and 25 µg/ml) lead to NO synthesis by macrophages in a dose-dependent manner (Figures 1A and 1B). Subsequent infection of MRP-primed macrophages with *L. major* did not affect NO production (Figure 1A). However, when cells were first infected and then stimulated with MRPs, NO production was reduced by around 35% (Figure 1B). To evaluate whether the effect of *Leishmania* infection was affecting iNOS protein levels, we performed western blotting. As expected, stimulation of macrophages with MRPs, led to an increase of iNOS expression (Figure 1C), and in MRP-primed macrophages followed by *Leishmania* infection, we observed increased of iNOS expression, which was maximal when 25 µg/ml of MRPs were added. At the same concentration of MRPs, the expression of iNOS was reduced when the cells were infected prior to stimulation (Figure 1C). These results revealed that *Leishmania* infection alters the capacity of MRPs to induce NO production by reducing iNOS expression.

### MRPs induce TNF-α production and *Leishmania* killing

**Figure 2:**
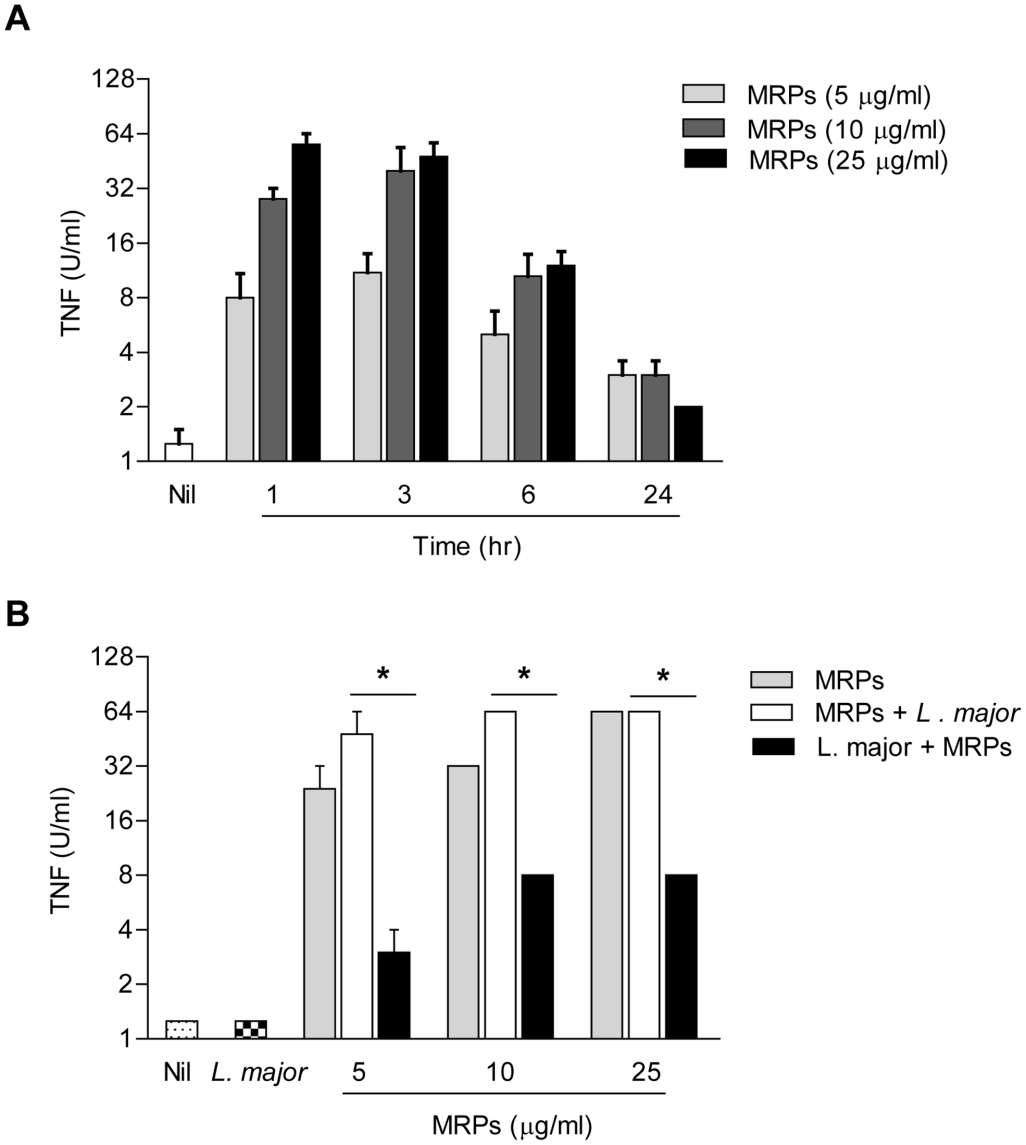
Effect of *Leishmania* infection on TNF-α production by MRPs. (**A**) B10R macrophages were stimulated with MRPs 8/14 (5, 10, 25 µg/ml) for 1, 3, 6 and 24 hr. TNF-α was measured in supernatants by TNF bioassay. (**B**) B10R macrophages were stimulated with MRPs 1 hr before or after infection with *L. major* and TNF-α was measured as in (**A**). (*) denotes *P*<0.05 between groups. Mean of three different experiments is shown.

Tumor necrosis factor α (TNF-α) is a multifunctional cytokine produced primarily by monocytes and macrophages. It has been shown that this cytokine is essential for the control of *Leishmania* at early stages of infection [20]. Therefore, we were interested in investigating whether MRPs were able to induce TNF-α production in macrophages. We performed a time and dose-dependent experiment, stimulating the cells for 1, 3, 6 and 24 hr with 5, 10 and 25 µg/ml of MRPs using TNF-sensitive L929 fibroblasts [21]. As shown in Figure 2A, the maximum peak of TNF-α production by MRP-stimulated macrophages occurred between 1 and 3 hr with 25 µg/ml of MRPs, decreasing thereafter. The time of 1 hr was chosen to evaluate the profile of TNF-α production during *Leishmania* infection. First, cells were primed for 1 hr with MRPs and then infected with *L. major*. Second, macrophages were infected with *L. major* overnight, followed by washes and stimulation with MRPs. As shown in Figure 2B, and similar to our NO data, MRP-primed macrophages subjected to *L. major* infection did not show altered capacity to produce TNF-α, however; the ability of *L. major*-infected cells to produce TNF-α in response to MRP stimulation was clearly reduced.

**Figure 3:**
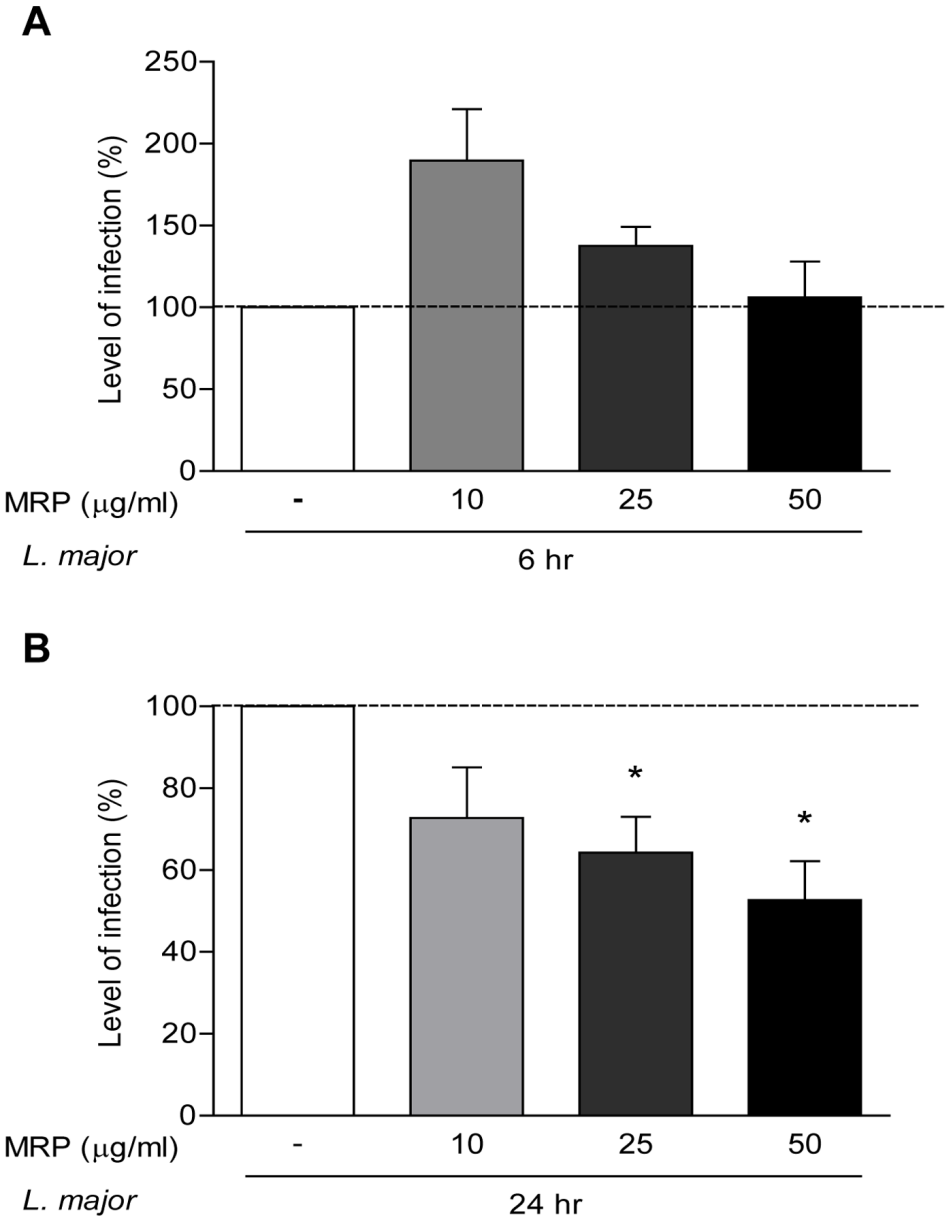
MRPs decrease the parasitic load in macrophages at 24*L. major*. B10R macrophages were primed with 5, 10 and 25 µg/ml of MRP8/14 for 1 hr, and then infected for 6 hr (**A**) and 24 hr (**B**) with *L. major* luciferase. Level of infection was measured by luciferase assay. The dashed line represents the infected cells at 6 hr (**A**) or 24 hr (**B**) without MRP treatment (RLU value = 100%). (*) denotes P<0.05 between the untreated 24 hr infected cells and cells primed with MRPs before 24 hr of infection. One representative experiment of three is shown.

MRP-priming conferred protection against *Leishmania* infection, as revealed by iNOS expression, NO and TNF-α production. Thus, we next evaluated whether this MRP-inducible microbicidal response correlated with an enhanced intracellular killing of the parasite [22]. To this end, macrophages were primed with various concentrations of MRPs prior to infection with a *L. major* strain expressing luciferase, then cells were collected at 6 and 24 hr post-infection. As shown in Figure 3A, at 6 hr post-infection, we observed a higher percentage of infection in the cells that were primed with 10 and 25 µg/ml of MRPs, compared to those that were not primed. However, after 24 hr of infection (Figure 3B) primed macrophages reduced the parasite load by 42% in a dose-dependent manner. Altogether these results could suggest that MRPs provide the cells with the ability to phagocytise and kill the parasites more efficiently that unprimed cells.

### MRPs induce MAPK phosphorylation and nuclear translocation of transcription factors

**Figure 4:**
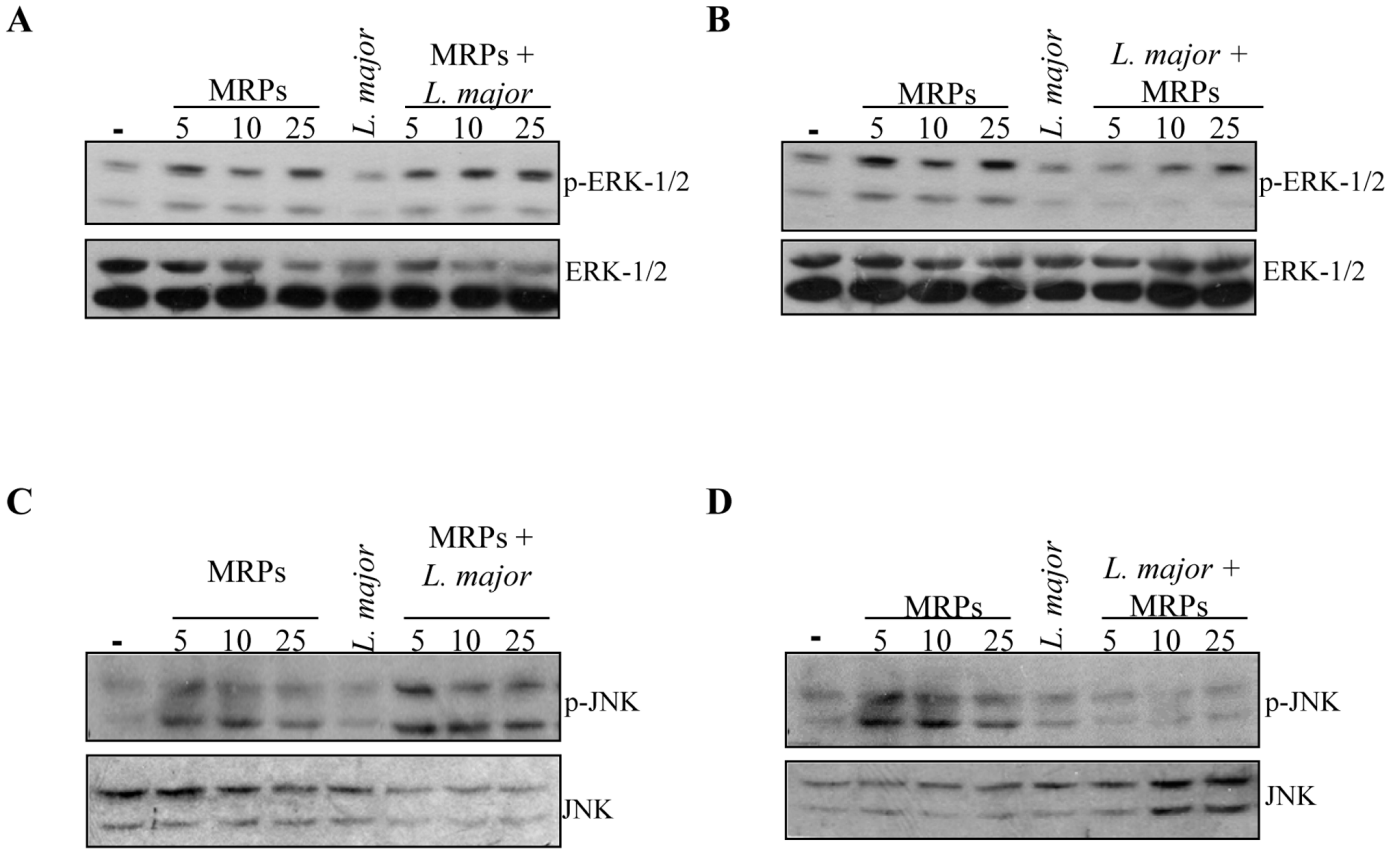
Impact of *Leishmania* infection on MRPs–induced ERK and JNK signaling. (**A**, **C**) B10R macrophages were only treated or primed with MRPs 8/14 (5, 10, 25 µg/ml) for 30 min before infection with *L. major* (1 hr). (**B**, **D**) B10R macrophages were only treated or MRP-stimulated (30 min) after 1 hr *L. major* infection. Protein lysates were assessed for phosphorylation of ERK 1/2 kinase (**A**, **B**) and JNK (**C**, **D**) kinase by immunoblotting. One representative experiment of three is shown.

As we observed that MRP stimulation increased the expression of iNOS, we further analyzed the signaling pathways involved in iNOS/NO production. We have previously reported that MRP-induced macrophage activation involves the participation of the ERK and JNK MAPKs [9]. Thus, it was critical to determine whether *Leishmania* could influence phosphorylation of these kinases in order to explain the incapacity of infected cells to respond to MRPs, knowing that *Leishmania* infection can interfere with signaling under the regulation of these kinases by activating host phosphatases [18], [23]. As expected, phosphorylation of both ERK and JNK (Figure 4) was observed in naive macrophages stimulated with MRPs. Phosphorylation of ERK and JNK was not altered in MRP-primed macrophages infected with *L. major* (Figures 4A and 4C). On the other hand, MRP-inducible ERK and JNK phosphorylation was strongly inhibited in *Leishmania*-infected cells (Figures 4B and 4D).

**Figure 5:**
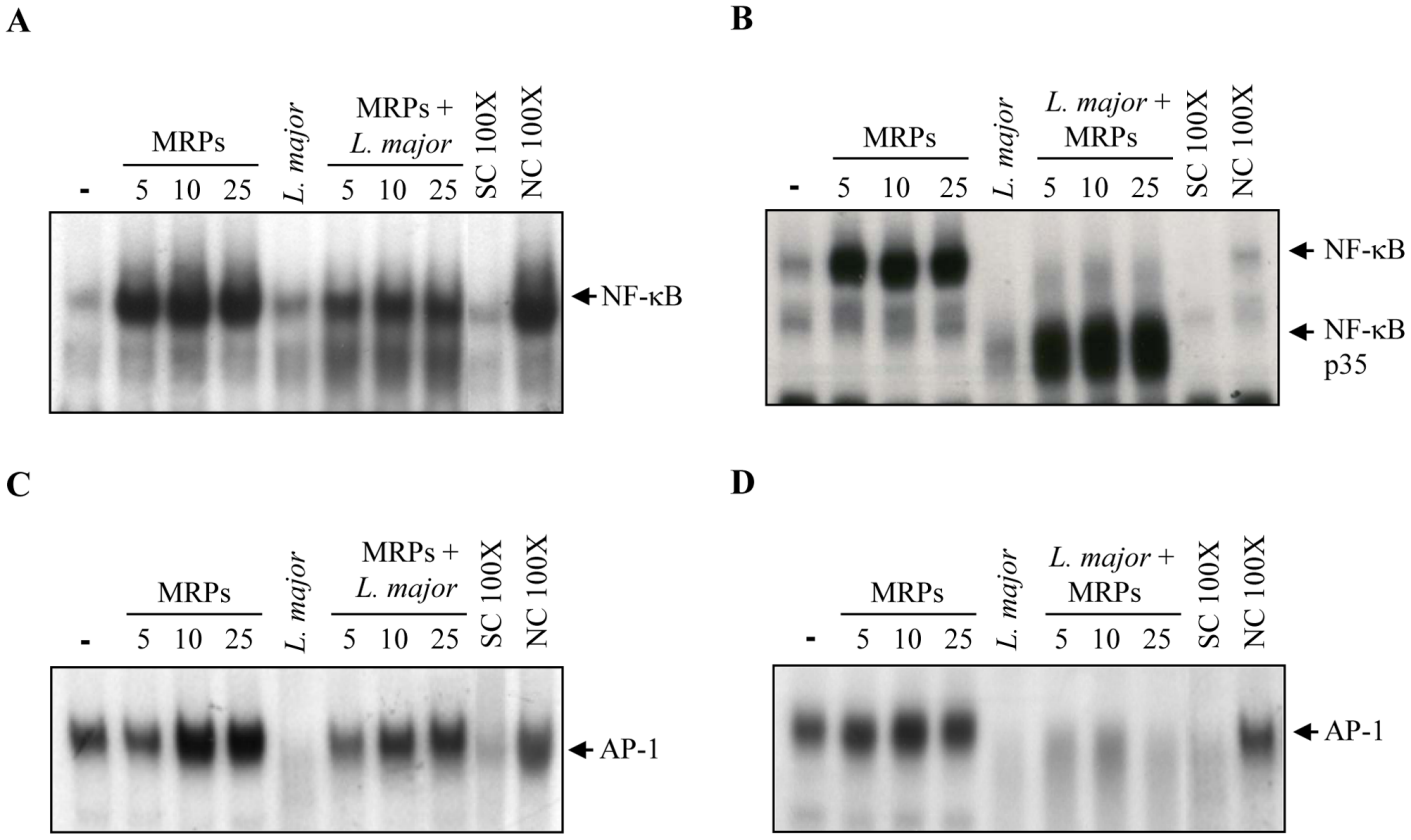
Nuclear translocation and binding of transcription factors (TFs). B10R macrophages were treated with MRPs 8/14 (5, 10, 25 µg/ml) for 1 hr or MRP-stimulated before infection with *L. major* (**A**, **C**) or treated 1 hr with MRPs after ON infection (**B**, **D**). Nuclear proteins were extracted and subjected to EMSA for NF-κB (**A**, **B**), and AP-1 (**C**, **D**). Consensus oligonucleotides non-specific competitors (Ins Co100×) and specific competitor (Spec Co100×) were used in and 100× molar excess. One representative experiment of three is shown.

To further characterize the activation of macrophage signaling after MRP stimulation, we investigated the nuclear translocation of transcription factors (TFs) involved in iNOS/NO production (e.g., NF-κB, STAT 1 and AP-1) by performing EMSA. A strong nuclear translocation of NF-κB (Figures 5A and 5B) and AP-1 (Figures 5C and 5D) occurred in response to MRPs stimulation. As shown in Figures 5A and 5C, MRP-primed macrophages showed translocation of NF-κB and AP-1 (Figure 5A). Nonetheless, NF-κB and AP-1 MRP-induced translocation was inhibited when macrophages were first infected with *Leishmania* (Figures 5B and 5D, respectively). In addition, it was possible to detect the p35 fragment (Figure 5B) that is a product of NF-κB degradation by *Leishmania* infection [24]. We also monitored the nuclear translocation of STAT; however, we did not observe any alteration of this TF in response to MRPs in either case (data not shown).

### *L. major* induces accumulation of leukocytes and MRP secretion in the air pouch

**Figure 6:**
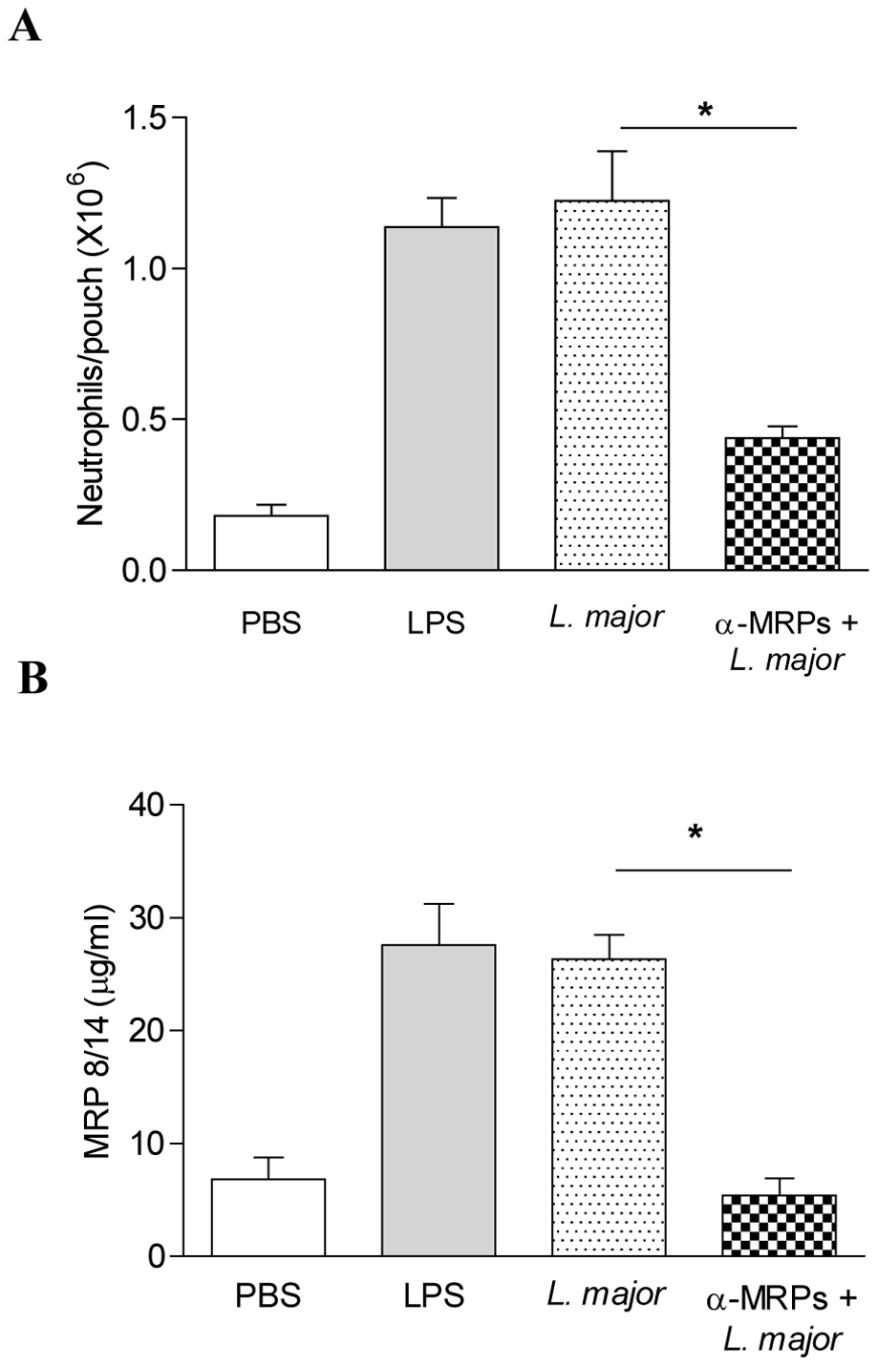
*Leishmania* induces leukocytes recruitment and MRP secretion in the murine air-pouch model. Air-pouches were raised in BALB/c mice during 6 days with sterile air, at day 7 they were stimulated with LPS or infected with *L. major* for 6 hr. Pouches were washed and total cell recruitment (**A**) and MRP secretion by ELISA were measured (**B**). BALB/c mice were treated with α-MRP 16 hr before infection with *L. major* for 6 hr. Total cell recruitment (**A**) and MRP secretion by ELISA (**B**) were measured. (*) denotes P<0.05 between the *L. major* infected group (n = 6) and the *L. major* infected group treated with α-MRPs.

Whereas MRPs modulate the microbicidal functions of macrophages *in vitro*, their role *in vivo* is still unknown. Therefore, using an air-pouch model we attempted to monitor this innate inflammatory event. Previous reports from our laboratory using this model have demonstrated that inoculation of *Leishmania* promastigotes led to the recruitment of inflammatory leukocytes at sites of injection within hours and this was accompanied by the secretion of various chemokines [25]. In addition, Tessier and collaborators have previously described that injection of LPS into the air-pouch induced neutrophil accumulation and the subsequent secretion of MRPs, reaching a maximum peak at 6 hr post-stimulation [2]. In this set of experiments, BALB/c mice were infected in the air-pouch with 10×10^6^ parasites for 6 hr. Afterwards, we evaluated the number of cells recruited and the secretion of MRPs. As shown in Figure 6A, *Leishmania* infection induced leukocyte recruitment comparable to LPS, neutrophils being around 80% of the total recruited leukocytes (Figure S1). In addition, *Leishmania* infection induced MRP 8/14 secretion by the recruited cells within the pouches (Figure 6B). To further monitor the implication of MRP secretion in the *Leishmania*-induced inflammatory cell recruitment, we neutralized MRPs using anti-MRP antibodies prior to infection with *L. major*. As shown in Figures 6A and 6B the use of these antibodies led to a significant reduction in cell recruitment, concomitantly with an almost complete abrogation of MRP secretion. It is important to point out that although we could still observe that the majority of the cells recruited in the mice injected with neutralizing antibodies were neutrophils (Figure S1), the total amount of recruited cells was significantly lower in these mice, compared to mice that received PBS, LPS or *L. major* (Figure 6A).

**Figure 7:**
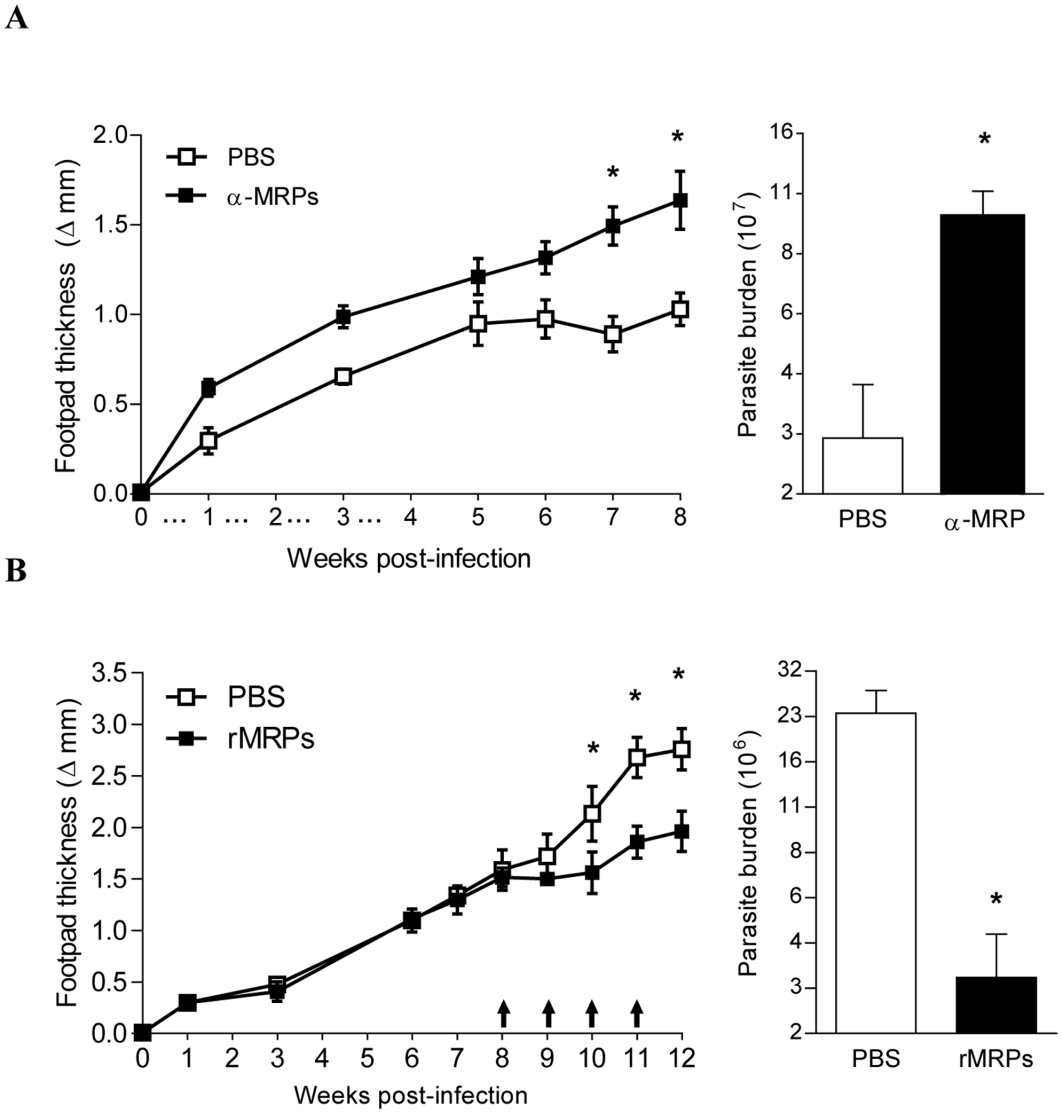
Immunological intervention with MRPs controls cutaneous Leishmaniasis. (**A**, **line graph**) BALB/c mice were injected with 2 mg of α-MRPs 8/14 one day before infection with *L. major* in the footpad and subsequent inoculation of the antibodies three times/week during four weeks after infection (…). Footpad sizes were measured every week for 8 weeks. (*) indicates significant difference P<0.01 between groups (n = 6). (**A, bar graph**) Parasites from footpads were extracted, and the parasitic burden was measured by limiting dilution assay, (*) denotes: P<0.05 between groups (n = 6). (**B, line graph**) BALB/c mice were injected with 10 µg of rMRPs 8/14 during 4 weeks (weeks 8 to12) directly into the infected footpad three times per week. Footpad sizes were measured every week for 12 weeks. (*) indicates significant difference P<0.01 between groups (n = 6). (**B, bar graph**) Parasites from footpads were extracted, and the parasitic burden was measured by limiting dilution assay, (*) denotes P<0.05 between groups (n = 6).

In the murine model, cutaneous Leishmaniasis is caused by injection of *L. major* or *L. mexicana* directly in the footpad. This model has been widely used to measure progression of infection in resistant and susceptible mice under different circumstances and for further isolation of parasites [26], [27]. To additionally investigate the role of MRPs during *Leishmania* infection, we monitored to which extend the neutralization of MRPs or the inoculation of recombinant MRPs would influence the progression of the infection *in vivo*. In a first set of experiments, we infected BALB/c mice and performed tri-weekly inoculation of MRP neutralizing antibodies for a period of 4 weeks. The progression of footpad thickening and development of lesion were followed over 8-weeks period. As shown in Figure 7A, mice that received anti-MRPs antibodies developed a significantly bigger footpad swelling during the first 8 weeks of infection comparatively to the untreated group. Significant differences were also detected between treated and control groups regarding the footpad parasitic load (Figure 7A, bar graph). These data suggest that MRPs secreted in the infectious environment could play an important role in the immunological events controlling Leishmaniasis development during the initial weeks of the infection.

To confirm the contribution of MRPs to the regulation of *Leishmania* infection, we tested whether recombinant MRP 8/14 (rMRP8/14) injected in infected footpads could lead to reduce *Leishmania*-related pathologies in mice. As reported in Figure 7B, BALB/c mice which started to receive inoculation of rMRPs at 8 week post-infection over a 4-weeks period, showed a clear and significant reduction of their footpad swelling and parasitic load comparatively to the control group (Figure 7B, bar graph).

**Figure 8:**
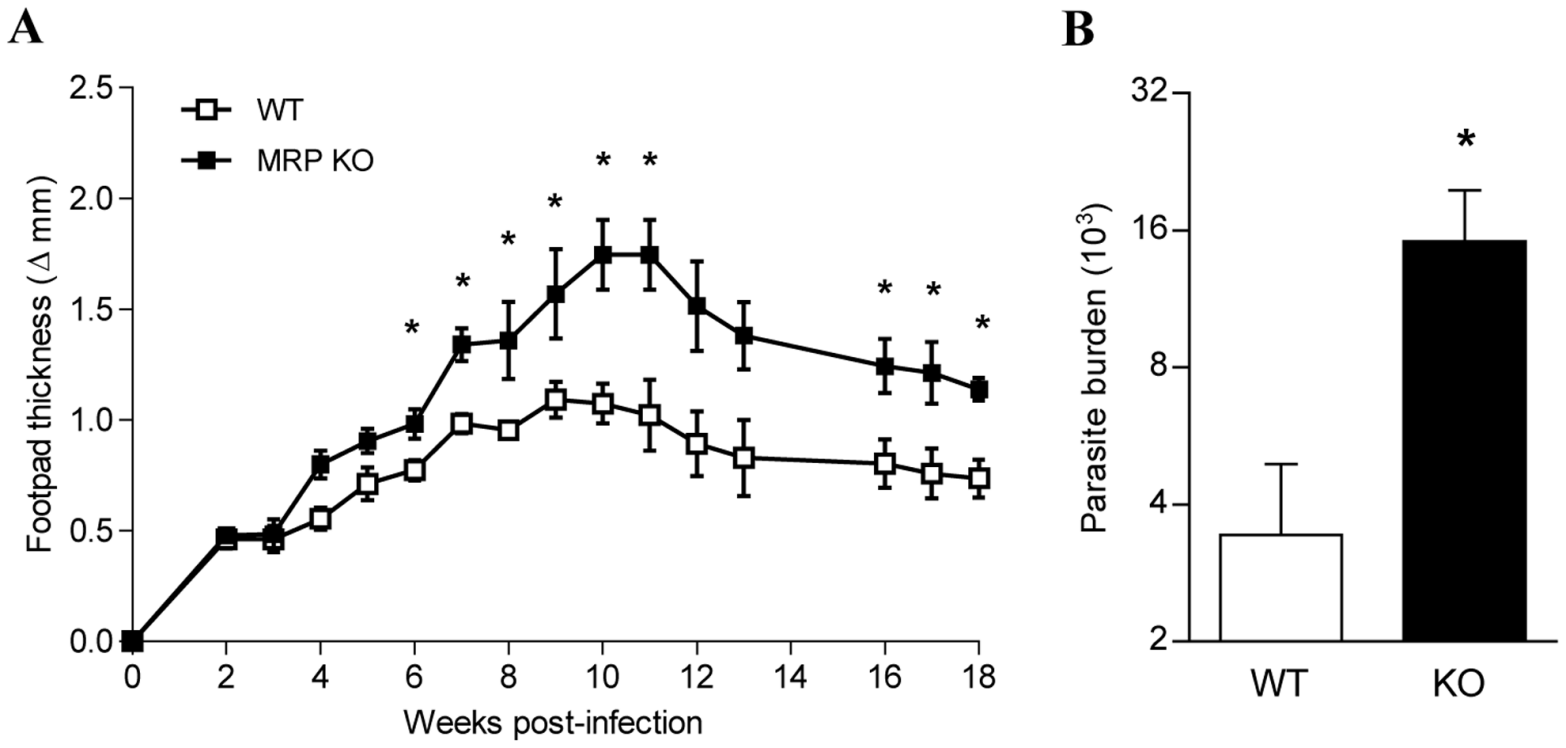
Progression of cutaneous Leishmaniasis in MRPs KO mice. (**A**): MRP14 KO mice (that also have reduced expression of MRP8) or C57Bl/6 wild type mice were infected with *L. major* in the footpad. Footpad sizes were measured every week for 18 weeks. (*) indicates significant difference P<0.01 between groups (n = 6). (**B**) Parasites from footpads were extracted, and the parasitic burden was measured by limiting dilution assay, (*) denotes: P<0.05 between groups (n = 6).

To further characterize the role of MRPs in the control of Leishmaniasis we used mice deficient for MRP14 that also fail to express MRP8 in peripheral and tissue leukocytes [28]. *L. major* infection caused a significantly greater pathology in MRP14 KO mice compared with its genetic background control (Figure 8A), as well as higher parasite load in the footpad after 19 weeks of infection (Figure 8B). This experiment was carried out for a longer period of time compared with the two previous experiments (using anti-MRPs or rMRPs) in order to observe the control of infection, as we used C57Bl/6 background mice. Although the parasite burden calculated by the limiting dilution assay was significantly lower in the C57BL/6 mice compared to the parasite burden from the BALB/c mice, we still observe that the absence of MRPs led to a higher parasite burden, even in resistant mice. This last set of experiments strongly suggests that MRPs play a significant role in the immunological mechanisms involved in the regulation of *Leishmania* infection. Moreover, these data unveil MRPs as potential therapeutic agents to treat Leishmaniasis.

## Discussion

MRP 8 and 14 also known as S100A8 and S100A9 belong to the S100 protein family, a large group of intracellular proteins associated with many cellular functions including contraction, motility, cell differentiation, calcium regulation among others [3]. In addition, the S100 proteins are also associated with different inflammatory diseases [29]–[32]. Recently, we and others have reported that MRP 8 and 14 can modulate macrophage functions including NO production [9]. Given that MRPs activate the macrophage signaling machinery and knowing that *Leishmania* parasites exert the opposite effect, we were interested in elucidating the role of MRP 8 and 14 during *Leishmania* infection both *in vitro* and *in vivo*.

Our results clearly showed that MRP-primed *Leishmania*-infected murine macrophages were able to produce NO with the concomitant expression of iNOS. These events correlated with a more efficient killing of the parasites as demonstrated by the luciferase assay. NO plays a key role in the macrophage microbicidal functions and is essential for the control of *Leishmania* infection [22]. In addition, we also found that MRP-primed macrophages produced high levels of TNF-α and were able to phosphorylate ERK and JNK kinases. More importantly, we observed that this priming resulted in an increased nuclear translocation of NF-κB and AP-1. This finding correlates with the fact that iNOS contains promoter binding sequences for these two transcription factors along with STAT1α [33].

The induction of MAPK phosphorylation and TFs nuclear translocation was observed very shortly after stimulation; the fact that MRPs are able to induce the NF-κB and the AP-1 pathways suggests that these TFs might act in synergy to enhance the expression of iNOS, resulting in high levels of NO and more efficient *Leishmania* killing. We have also reported that MRPs are recognized by Toll like receptor 4 (TLR4) [9]. This is in line with the observation that NF-κB is strongly induced by MRPs, on the other hand, efficient induction of AP-1 might be due to the fact that ERK and JNK are up-stream activators of c-Jun and c-Fos which dimerize to form active AP-1 complexes [34].

Additionally, we observed that macrophages that were first infected and then stimulated with MRPs, did not have the capacity to respond in the same way as primed macrophages, since the levels of NO and TNF production as well as the phosphorylation of JNK and ERK and the nuclear translocation of TFs were substantially reduced. This suggests that the parasite is able to abrogate the activation of the macrophage signaling machinery induced by MRPs in order to survive inside the host. One of the main mechanisms adopted by the parasite to subvert the immune response is the rapid activation of host phosphatases [18], [23], [35]. This fact might explain the poor MAPK phosphorylation and TFs nuclear translocation observed in macrophages first infected and then stimulated with MRPs.

Studies made by our group have shown that mouse infection with *Leishmania* parasites in the air-pouch model leads to neutrophil recruitment [25], Here, we demonstrated that MRPs controlled neutrophil recruitment induced by *Leishmania* or LPS. However, the exact role of neutrophils during cutaneous Leishmaniasis is still controversial. For instance Lima et al. [36] showed that there is a massive infiltration of neutrophils soon after skin infection with *L. major*, they investigated in more detail the role of neutrophils in resistant C57BL/6 and susceptible BALB/c mice by depleting neutrophils with specific antibodies. They showed that neutrophil depletion in both susceptible and resistant mice accelerated parasite spreading and caused more severe footpad swelling. These data suggested that neutrophils are of crucial importance in early control of parasite infection. In contrast, a study made by Laskay et al. [37] showed that *Leishmania* uses neutrophils as an evasion strategy, since the parasite survives inside these cells and use them as “Trojan horses” to get access into the macrophages where it will survive and multiply. Later, the same group showed that *Leishmania*-infected neutrophils also are uptake by dendritic cells inhibiting early immune response against *Leishmania* in the tissue [38]. Some other reports have shown that depletion of neutrophils in BALB/c mice inhibited the IL-4 response and promoted partial resistance [39]. Using B10R macrophages we also observed significantly increase of parasite infection when cells were treated with MRPs, however, it did not reflect in the survival of the *Leishmania* within macrophages (Figure 3).

More recently, Peters et al. [40], [41] showed that depletion of neutrophils reduces the ability of the parasite to establish productive infections. Furthermore, they reported that the neutrophils are the initial host cell for a substantial fraction of parasites and that there is more control of the infection when the neutrophils are not present. Interactions between cellular populations have been pointed out as important to either the control or development of the disease, and one of the most important cell interactions is the one between neutrophils and macrophages. It has been demonstrated by some groups that interactions of apoptotic or necrotic neutrophils with macrophages may interfere with the outcome of the infection. Interaction of dead neutrophils with *L. major*-infected peritoneal macrophages isolated from BALB/c mice, led to an increase in parasite growth, a mechanism mediated by the TFG-β and PGE2 produced by macrophages; however, macrophages isolated from resistant C57BL/6 mice and co-cultured with the same dead neutrophils presented a good microbicidal activity, mediated by TNF-α, therefore controlling the infection [42]. Concurring with this, Afonso et al. demonstrated that phagocytosis of apoptotic neutrophils by *L. amazonensis*-infected macrophages led to an increase on the parasite burden; however, phagocytosis of necrotic neutrophils resulted in parasite killing in a NO-independent manner, but dependent on ROIs [43]. Later on, it was observed that elastase produced by neutrophils plays a key role in the control of the infection, since this molecule activates the microbicidal mechanisms of the *L. major*-infected macrophages in a TLR-4-dependent manner [44].

It has also been studied that, depending on the infecting *Leishmania* species, or even the specific strain, the interaction between neutrophils and macrophages can lead to resistance or susceptibility to the infection. For instance, Novais F. *et al*, demonstrated that *L. braziliensis* elimination depends on the interaction between neutrophils and macrophages, in a TNF-dependent mechanism [45]. The same effect was observed in the control of *L. amazonensis*, where it was shown that TNF-α, elastase and platelet activation factor, produced by neutrophils, were responsible for parasite killing. On the other hand, this study also showed that NO and ROIs were not involved in the clearance of the parasite, as has been observed with other *Leishmania* species [46]; we consider that, in both cases, it is also possible that neutrophils secrete MRPs, and this secretion helps to control the infection. Contrary to what was shown by Ribeiro *et at.*, it has been demonstrated that *L. major*-infected macrophages isolated from C57BL/6 resistant mice induce apoptosis of neutrophils, therefore favoring the propagation and survival of the parasite [47].

Our data are partially in agreement with some of the previous observation, since we clearly demonstrated that there is neutrophil recruitment at the site of infection in the air-pouch model; moreover, we showed that these neutrophils are able to secrete MRPs and that depletion of these MRPs significantly reduced the amount of recruited neutrophils and consequently MRP secretion. In addition, infection with *L. major* in the footpad of susceptible BALB/c mice and depletion of MRPs resulted in an increased parasite load and footpad swelling. These results strongly suggest that MRPs are important to control *Leishmania* infection. Strengthening this fact, when we treated mice with rMRPs directly in the footpad, we observed a significant reduction in size of lesion and parasite load. A potential mechanism underlying these events could be that injection of MRPs leads to an enhanced neutrophil recruitment, which in turn, can secrete more MRPs creating a positive feedback loop of constant secretion of MRPs, where it is possible that neutrophils are actually containing the progression of the infection, concomitant with the fact that these proteins present by themselves antimicrobial properties [6]. Additionally, we do not rule out the possibility that direct injection of MRPs also induced monocyte recruitment and as observed in our *in vitro* results which showed that MRP-primed macrophages are able to produce high levels of NO, being this responsible for the killing and more efficient control of the infection. However, whether the control of the infection *in vivo* is NO-mediated needs further investigation.

In summary our data showed for the first time that MRP 8 and 14 play an important role in the control of *Leishmania* infection *in vivo* and *in vitro* and support the idea that they could have a potential role as therapeutic drugs.

## Supporting Information

Figure S1 **Differential count of cells recruited into the air-pouch.** Differential count of cells obtained after washing the raised air-pouches in BALB/c mice inoculated with PBS, LPS, *L. major* or anti-MRP + *L. major*. Cells were stained with diff-quick stain and counted in a bright field microscope. 300 cells were counted by sample.
(TIF)
