# Supplementary figures and images for: Impact of Neutrophil-Secreted Myeloid Related Proteins 8 and 14 (MRP 8/14) on Leishmaniasis Progression
Source: PLoS Negl Trop Dis. 2013 Sep 26;7(9):e2461. doi: 10.1371/journal.pntd.0002461 (PMC3784490; doi:10.1371/journal.pntd.0002461)

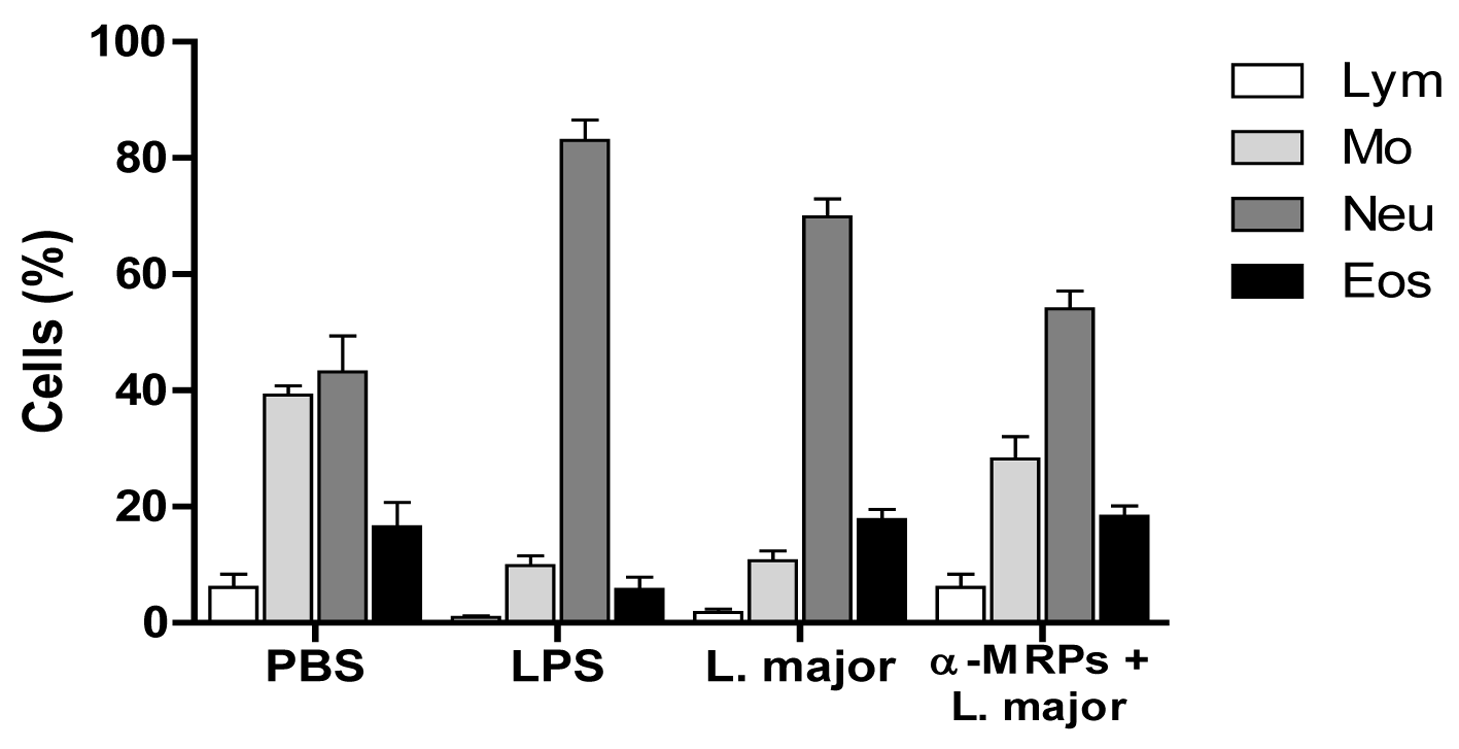

Supplement: Figure S1 — Differential count of cells recruited into the air-pouch. Differential count of cells obtained after washing the raised air-pouches in BALB/c mice inoculated with PBS, LPS, L. major or anti-MRP + L. major. Cells were stained with diff-quick stain and counted in a bright field microscope. 300 cells were counted by sample. (TIF) [file pntd.0002461.s001.tif]
